# *FZD6* is a novel gene for human neural tube defects

**DOI:** 10.1002/humu.21643

**Published:** 2011-02

**Authors:** Patrizia De Marco, Elisa Merello, Andrea Rossi, Gianluca Piatelli, Armando Cama, Zoha Kibar, Valeria Capra

**Affiliations:** 1 Neurosurgery Department, G. Gaslini InstituteGenova, Italy; 2 Neuroradiology Department, G. Gaslini, InstituteGenova, Italy; 3 Department of Obstetrics and Gynecology, CHU Sainte Justine Research Center and University of MontrealMontreal, Québec, Canada

**Keywords:** neural tube defects (NTD), planar cell polarity pathway, *FZD6*

## Abstract

Neural tube defects (NTDs) are severe malformations of the central nervous system, affecting 1 of 1,000 live births. Mouse models were instrumental in defining the signaling pathways defective in NTDs, including the planar cell polarity (PCP), also called noncanonical Frizzled/Disheveled pathway. Based on the highly penetrant occurrence of NTDs in double *Fzd3/Fzd6^−/−^* mutant mice, we investigated the role of the human orthologues, *FZD3* and *FZD6*, by resequencing a cohort of 473 NTDs patients and 639 ethnically matched controls. While we could not demonstrate a significant contribution of *FZD3* gene, we identified five rare *FZD6* variants that were absent in all controls and predicted to have a functional effect by computational analysis: one de novo frameshift mutation (c.1843_1844insA), three missense changes (p.Arg405Gln, p.Arg511Cys p.Arg511His), and one substitution (c.^*^20C>T) affecting the 3′-untranslated region (UTR) of the gene. The overall rate of predicted deleterious variants of *FZD6* was 5.1-fold higher in cases compared to controls, resulting in a significantly increased NTDs mutation burden. This study demonstrates that rare nonsynonymous variants in *FZD6* may contribute to NTDs in humans and enlarges the spectrum of mutations that link PCP pathway to NTDs. Hum Mutat 33:384–390, 2012. © 2011 Wiley Periodicals, Inc.

## Introduction

Neural tube defects (NTDs; MIM# 182940) are congenital malformations due to the incomplete closure of the neural tube during the early development. NTDs are known to occur in 1 of every 1,000 pregnancies, with varying rates reported among the world populations [Botto et al., 1999; Copp et al., 2003]. The most common NTDs are anencephaly, which results from failure of fusion of the cranial neural tube, and myelomeningocele (commonly called spina bifida), which results from failure of fusion of neural tube in the spinal region. A relatively rare form of NTDs, known as craniorachischisis, results from failure of neural tube closure along the entire body axis. Anencephaly and myelomeningocele are referred as "open" NTDs because the affected regions are exposed to the body surface. There are also a number of closed or skin-covered conditions that involve the neural tube, including encephalocele, and meningocele, lipomyelomeningocele, also referred to as spina bifida occulta, and caudal regression [Rossi et al., 2004].

NTDs have a complex etiology involving both environmental and genetic factors. Although folic acid, supplemented in the mother periconceptionally, appears to have dramatically reduced the frequency of NTDs [Czeizel and Dudás, 1992; MRC Vitamin Study Research Group, 1991], the mechanism by which folate deficiency predisposes to NTDs remains unclear.

A major cellular event occurring during neurulation is convergent extension, a morphogenetic process by which the presumptive notochord and neural plate lengthen and narrow due to the mediolateral intercalation of cells [Ueno and Greene, 2003; Wallingford et al., 2002]. During this process, cells elongate mediolaterally and produce polarized cellular protrusions that enable them to move directionally and to intercalate to other neighboring cells [Keller et al., 2000; Wallingford and Harland, 2002]. Convergent extension is mediated by the highly conserved planar cell polarity (PCP) pathway, also called the noncanonical Frizzled-Dishevelled signaling cascade. Core PCP genes include: *Strabismus*/V*an Gogh* (*Stbm/Vang*), *Frizzled* (*Fz*), *Dishevelled* (*Dsh*), *Flamingo* (*Fmi*), *Prickle* (*Pk*), and *Diego* (*Dgo*) [Simons and Mlodzik, 2008]. Evidence for the involvement of the PCP pathway in convergent extension in vertebrates has emerged from studies of a wide range of mutants of orthologs of PCP genes in several animal models such as zebrafish, *Xenopus,* and mouse [Barrow, 2006; Heisenberg et al., 2000; Montcouquiol et al., 2006; Tada and Smith, 2000; Wallingford et al., 2000; Wallingford, 2005]. Recently, we identified mutations in two human PCP genes, *VANGL1* and *VANGL2*, providing evidence for a pathogenic role of PCP signaling in human NTDs [Kibar et al., 2007, 2009, 2011]. Other PCP genes have a potential role in convergent extension movements and neural tube closure in normal and abnormal neurulation, and hence need to be studied in large human NTDs cohorts.

Frizzleds are seven-pass transmembrane (7TM) receptors that transduce critical cellular signals during development. Frizzleds share a cysteine-rich domain (CRD) in the N-terminal extracellular region, which binds several secreted proteins, and among them, proteins of the Wnt family [Schulte and Bryja, 2007]. Amino acid hydropathy analysis predicts a conventional 7TM structure, three extra- and three intracellular loops, and an intracellular carboxy-terminal PDZ (Postsynaptic density 95 [PSD-85]; Discs large [Dlg]; Zonula occludens-1 [ZO-1]) domain involved in protein interaction [Schulte and Bryja, 2007]. Three main signaling pathways are activated by Frizzleds: the PCP pathway, the canonical Wnt/β-catenin pathway, and the Wnt/calcium pathway [Schulte and Bryja, 2007]. Wnt signaling is important for cell division (proliferation), attachment of cells to one another (adhesion), cell movement (migration), and many other processes before and after birth [Umbhauer et al., 2000; Wang et al., 1996]. It is widely accepted that binding of the phosphoprotein Disheveled (Dvl/Dsh) and its membrane recruitment by Frizzled is the critical event in Frizzled-induced signal transduction for all three Frizzled signaling pathways [Cong et al., 2004]. There are currently 11 mammalian Frizzled proteins [Schulte and Bryja, 2007], which have been implicated in a variety of developmental processes, including several that involve the nervous system. *Fzd3* is required for axonal outgrowth and guidance in the central nervous system (CNS) [Wang et al. 2002, 2006b]. Targeted deletion of the mouse *Fzd3* gene leads to a curled tail phenotype and flexed hindlimbs in newborns, and cephalic neural tube closure in a small percentage of embryos [Wang et al. 2002, 2006a]. Double mutants *Fzd3^−/−^/Fzd6^−/−^* embryos exhibit craniorachischisis (a fully open neural tube) and curled-tail with a 100% penetrance, a partially penetrant failure of eyelid closure, and misorientated auditory and vestibular sensory hair cells, providing the most direct evidence for a functional connection between PCP components and mammalian tissue fusion processes [Wang et al., 2006a]. Expression studies in humans have shown that *FZD3* (MIM# 606143) and *FZD6* (MIM# 603409) are widely expressed in both embryonic and adult tissues, including brain and CNS [Sala et al., 2000; Tokuhara et al., 1998].

The role of PCP signaling in the pathogenesis of NTDs in animal models and humans as well as the role of *Fzd3 and Fzd6* in neural tube closure in mouse models prompted us to investigate if the human orthologs *FZD3* and *FZD6* genes could play a role in the pathogenesis of human NTDs, by resequencing these genes in a large cohort of patients and controls.

## Subjects and Methods

### Patients and Controls

The patients cohort consisted of (1) 391 Italian patients recruited at the Spina Bifida Center of the Gaslini Hospital in Genova, Italy; among them, 284 have already been included in our previous studies [Kibar et al., 2007, 2009, 2011]; (2) 82 patients recruited at Sainte Justine Hospital in Montreal, Canada. Detailed clinical information on both cohorts is presented as Supp. Table S1. All patients had isolated nonsyndromic NTDs. Around 19% of patients had a positive family history documented by clinical records (MRI and X-ray images).

The control group comprised 433 Italian individuals consisting of randomly selected children admitted to the Gaslini Children's Hospital for miscellaneous illnesses and healthy young adults who contributed samples to the blood bank of the Gaslini Institute. The samples were anonymous and information associated with these samples included only sex, region of birth, and age. The control group also included 206 Caucasian controls of French–Canadian ancestry. All control individuals (*N* = 639) were seen by a neurologist and confirmed to be healthy. Samples from patients and controls were collected with the approval of the Local Ethics Committees and written informed consent was obtained from all participating patients, parents, and controls.

### Resequencing and Genotyping

**Figure 1:**
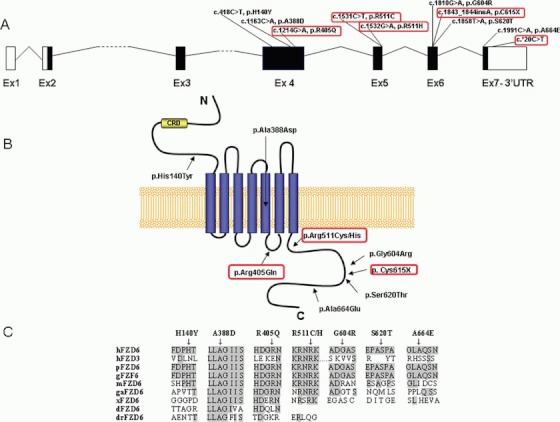
Rare nonsynonymous *FZD6* variants. **A:** Schematic representations of *FZD6* (RefSeq: NG_ 028909.1; NM_001164615.1) gene with the approximate locations of the identified rare nonsynonymous variants. *FZD6* mutations absent in controls and predicted to have a functional effect are circled in red. The DNA mutation numbering is according to cDNA numbering with nucleotide +1 as the A of the ATG translation initiation codon in the reference sequence. Ex, exon**. B:** Topological model of the *FZ6* protein. The approximate positions of the mutations identified are shown. Nonsynonymous *FZD6* mutations absent in controls and predicted to have a functional effect are circled in red. CRD, cysteine-rich domain. **C:** Clustal W protein sequence alignment of human FZD6 with human FZD3 and orthologues from other species. Residues conserved between FZD and other family members are gray highlighted. Ensemble accession numbers of human FZD6 (hFZD6): ENSP000004290551; human FZD3 (hFZD3): ENSP000002400931, *Pan troglodytes* FZD6 (pFZD6): ENSPTRP000000437521, *gorilla* FZD6 (gFZD6): ENSGOP000000153811; *mouse* FZD6 (mFZD6): ENSMUSP000000229061, *gallus* FZD6 (gaFZD6): ENSGALP000000258431, *Xenopus* FZD6 (xFZD6): ENSXETP00000088811, *Danio Rerio* FZD6 (dFZD6): ENSDARP000000627021, and *Drosophila* FZD6 (drFZD6): FBpp00754851.

Genomic DNA was isolated from EDTA peripheral blood samples, by using the QIAamp DNA blood Kit (Qiagen, Milan, Italy), according to the manufacturer's protocol. The genomic structures of human *FZD3* and *FZD6* were determined using the NCBI GenBank (*FZD3***:** NG_029723.1 and NM_017412.3; *FZD6*: NG_028909.1 and NM_001164615.1) (Fig. 1; Supp. Fig. S1). The whole-coding region of *FZD3* (3,933 bp long) and *FZD6* (3,806 bp long) with about 180 bp of the 5′-untranslated region (UTR) and 150 bp of the 3′ UTR were amplified. The portions of the introns that were sequenced ranged from 120 to 160 bp with an average of almost 140 bp. Polymerase chain reaction (PCR) was carried out using the AmpliTaqGold (Applied Biosystems, Monze, Italy) as per manufacturer's instructions. Direct dye terminator sequencing of PCR products was carried out using the ABI Prism Big Dye Systems (Applied Biosystems). Samples were run on ABI 3700 automated sequencer (Applied Biosystems) and analyzed using the PhredPhrap software 5.04 (http://droog.gs.washington.edu/polyphred). Nonsynonymous changes that were most likely expected to have a detrimental effect were further genotyped using the iPLEX^TM^ Gold assay for SNP Genotyping (Sequenom, San Diego, CA) [Ehrich et al., 2005]. For variants identified in NTDs cases, we tested the cosegregation by sequencing the corresponding fragment in available additional family members.

### Bioinformatics

Mutations were annotated according to the HGVS nomenclature (http://www.hgvs.org/mutnomen). Nucleotide numbering reflects cDNA numbering with +1 corresponding to the A of the ATG translation initiation codon 1 in the reference sequence, according to journal guidelines. A variant was designated as novel if it was not found in either dbSNP Build 133 or in the 1,000 Genome Project (release of May 2011). The potential pathogenic effect of the missense mutations on protein function was predicted using two software programs: PolyPhen (Polymorphism Phenotyping) (http://genetics.bwh.harvard.edu/pph/) and PANTHER (Protein Analysis Through Evolutionary Relationships) (http://www.pantherdb.org/). Possible effects on the secondary structure of the protein were assessed by Dompred software (http://bioinf.cs.ucl.ac.uk/dompred/) that is designated to predict putative protein domains and their boundaries. We used an input *E*-value cutoff default of 0.01 and the number of PSI-BLAST interactions at the default of 5. Multiple alignments of the FZD3 and FZD6 proteins were done using the CLUSTAL W program, freely available online (http://npsa-pbil.ibcp.fr). Localization of the variants in protein domains was assessed by Uniprot (http://www.uniprot.org/). Potential effect of the 3′ UTR variants by altering MicroRNAs (miRNA) binding sites was evaluated by MicroRNA.org site (http://www.microrna.org) that uses the mirSVR regression model for target site predictions (mirSVR score ≤ −0.1).

### Association Analysis

Rare variants were defined as those having a minor allele frequency (MAF) of at most 1%. Chi-square analysis and Fisher's exact test (two-tailed) was used to test for association with the variants.

## Results

The resequencing analysis of all 1,112 subjects identified a total of 12 common variants (MAF ≥ 1%) and 28 rare variants (MAF < 1%), of which 22 were never reported before. No variants that affect highly conserved consensus splice sites were identified (Supp. Table S2). All of the common variants were known single nucleotide polymorphisms (SNPs) and none were associated with NTDs (*P* > 0.05;) (Supp. Table S3). Rare variants were overrepresented in patients compared to controls (34 patients/14 controls; *P* = 0.0001; Table 1 and Supp. Table S4). No NTD patient or control was a carrier of rare missense mutations in both *FZD3* and *FZD6*.

**Table 1:** Novel and Known Rare Variants in the Coding and Flanking Intronic Sequence of *FZD6* Gene

Nucleotide change^a^ (rs ID)	Amino acid change	Resequencing 473 pt/639 ct	Amino acid conservation	Dompred Prediction^b^	PANTHER prediction (subPSECscore)^c^	PolyPhen prediction (PSIC score)^d^	mirSVR regression model (mirSVR score)^e^
c.374+7A>C	-	1/0	-	-	-	-	-
c.418C>T(rs80216383)	p. His140Tyr	2/1^f^	No	Random coiled, alteration	Deleterious (−4.05)	Possibly damaging (2.11)	-
c.765C>T	p.Gly255Gly	3/0	Yes	Random coiled, -	-	-	-
c.1125G>A	p.Leu374Leu	1/0	Yes	Alpha-helix, -	-	-	-
c.1163C>A	p. Ala388Asp	1/2	Yes	Alpha-helix,alteration	Deleterious (−5.52)	Possibly damaging (1.92)	-
**c.1214G>A**	**p.Arg405Gln**	**2/0**	**Yes**	**Random coiled,alteration**	**NA**	**Benign (0.38)**	**-**
c.1392+67A>T	**-**	1/0	**-**		**-**	**-**	**-**
c.1392+115C>T	**-**	1/0	**-**		**-**	**-**	**-**
c.1393-109G>T	**-**	1/0	**-**		**-**	**-**	**-**
**c.1531C>T**	**p.Arg511Cys**	**1/0**	**Yes**	**Random coiled,alteration**	**Not deleterious (**−**2.47)**	**Possibly damaging (2.45)**	**-**
**c.1532G>A**	**p.Arg511His**	**1/0**	**Yes**	**Random coiled,alteration**	**Deleterious (**−**3.65)**	**Benign (0.15)**	**-**
c.1809C>T	p.Asp603Asp	1/0	No	Random coiled, -	-	-	-
c.1810G>A(rs79408516)	p.Gly604Arg	2/1^f^	No	Random coiled, no alteration	Not deleterious (−1.99)	Benign (0.004)	
**c.1843_1844insA**	**p.Cys615X**	**1/0**	**Yes**	**-**	**-**	**-**	**-**
c.1858T>A(rs116195528)	p.Ser620Thr	2/1^f^	No	Random coiled, no alteration	Not deleterious (−1.02)	Benign (0.01)	-
c.1991C>A(rs12549394)	p.Ala664Glu	2/2	No	Random coiled, no alteration	NA	Benign (0.35)	-
c.^*^20C>T	**-**	**1/0**	**-**	**-**	**-**	**-**	**Probably alterating miRNA binding site (**−**0.6)**
Rare variants		24/7					
Rare functional deleterious variants		9/3 (*P* = 0.036)					

a FZD6 GenBank RefSeq number NM_001164615.1 and NG_028909.1. Nucleotide numbering reflects cDNA numbering with +1 corresponding to the A of the ATG translation initiation codon 1 in the reference sequence.

b Location in protein secondary structure/potential structure alteration.

c subPSEC score: substitution position-specific evolutionary conservation score. Continuous values range from 0 (neutral) to about −10 (most likely to be deleterious). −3 is the cutoff point.

d PSIC (**p**osition-**s**pecific **i**ndependent **c**ounts) score: <1 benign, >1 possibly damaging, >2 probably damaging.

e mirSVR is a regression model that computes a weighted sum of a number of sequence and context features of the predicted miRNA–mRNA duplex. mirSVR downregulation scores are calibrated to correlate linearly with the extent of downregulation and therefore enable accurate scoring of genes with multiple target sites by simple addition of the individual target scores (mirSVR cut off score ≤ −0.1). The mutation altered the binding site for MIR628.

f These two patients and one control individual are carriers of three *FZD6* in cis mutations: p. His140Tyr , p.Gly604Arg, p.Ser620Thr.

pt, patients; ct, controls; NA, not aligned; 3′UTR, 3′-untranslated region. Deleterious variants not found in controls are in bold.

To identify mutations that cause or predispose to NTDs, we focused our analysis on 13 (three of *FZD3* and 10 of *FZD6*; Supp. Table S2) rare nonsynonymous (missense, frameshift, and 3′ UTR) variants as these were more likely to have a substantial effect on protein function. A multifaceted computational approach based on the functional prediction of nonsynonymous variants, the conservation of the altered amino acid, the effect on the secondary protein structure, and alteration of miRNAs-binding site for noncoding 3′ UTR variants was used to highlight variations potentially involved in NTDs.

Resequencing of the *FZD3* gene identified only one possibly damaging mutation, suggesting that this gene does not play a major role in the causation of NTDs (Supp. Table S4 and Supp. Fig. S1).

### Rare Variants in *FZD6* Gene

We identified five rare *FZD6* variants that were absent in all controls and predicted to have a functional effect. One of them is a de novo frameshift mutation that introduces a premature stop codon (p.Cys615X [c.1843_1844insA]), three are missense changes (p.Arg405Gln [c.1214G>A], p.Arg511Cys [c.1531C>T], p.Arg511His [c.1532G>A]), and last one is a single nucleotide substitution (c.^*^20C>T), affecting the 3′ UTR of the *FZD6* gene. (Fig. .1; Table 1). Nevertheless, the overall rate of predicted deleterious variants was 5.1-fold higher in cases compared to controls, for significantly increased mutation burden with NTDs (nine patients/three controls; *P* = 0.036).

**Figure 2:**
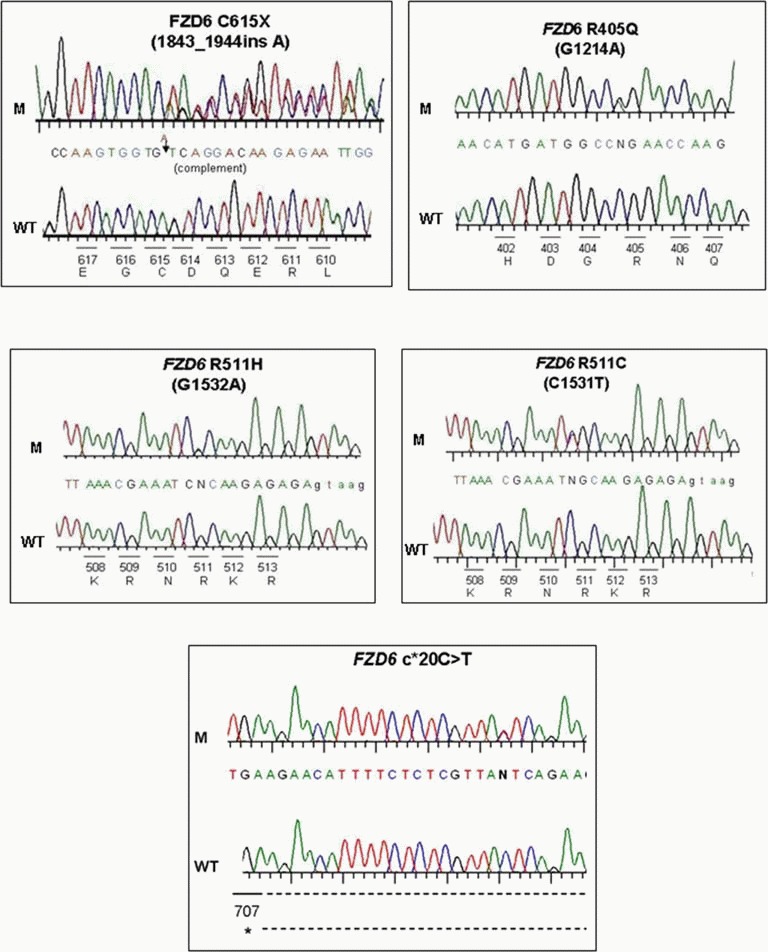
Electropherograms of nonsynonymous *FZD6* mutations absent in controls and predicted to have a functional effect. The altered amino acids with their positions and corresponding nucleotide changes (capital letters) are shown with the sequencing trace data. Small letters indicate intronic sequence. The *FZD6* p.Cys615X mutation is shown in the 3′→5′ direction and the complement nucleotide is shown. The other mutations are shown in sense (5′→3′) direction. WT, wild-type sequence; M, mutated sequence. ^*^, stop codon.

The *FZD6* p.Cys615X (c.1843_1844insA) was a de novo mutation (absent in the parents), deriving from the insertion of an A nucleotide at position 1,843 of the coding region that introduces a premature stop codon at position 615 (Figs. 1A, B, and 2). It was identified in an Italian patient affected by a complex dysraphism consisting of anterior thoracic meningocele, hydromyelia, intradural lipoma, scoliosis, and multiple vertebral and costal anomalies (Table 2). Maternity and paternity were confirmed by microsatellite markers analysis. The p.Cys615X mutation encodes for a truncated protein that lacks the last 51 amino acids in its carboxyl-terminal tail.

**Table 2:** Clinical Features of NTDs Patients Carrying Unique FZD6 Mutations

Sex, ethnic group	Mutations	NTDs type	Inheritance	Clinical features
F, C	p.Arg405Gln	Spinal, open	Father carrier	Lumbo-sacral MMC, hydrocephalus. Familial case: maternal third-degree relative has open NTD; parents are first-degree cousins
M, C	p.Arg405Gln	Spinal, open	UN (parents NA)	MMC, sphincteric incontinence, neurogenic bladder.
F, C	p.Arg511Cys	Spinal, open	UN (parents NA)	MMC, Chiari malformation type II
M, C	p.Arg511His	Spinal, closed, complex	Mother carrier	CRS: sacral agenesis, spondilo-costal dysplasia, MC, tethered cord, hydromyelia, club foot, left kidney agenesis, convex dorso-lumbar scoliosis
F, C	p.Cys615X	Spinal, closed, complex	No, de novo	Right anterior thoracic MC, hydromyelia, intradural lipoma, spondilo-costal dysplasia, complex scoliosis
M, C	c.^*^20C>T	Spinal, open	UN (father UN)	Lumbo-sacral MMC, Chiari malformation type II, hydrocephalus

MMC, myelomeningocele; MC, meningocele; CRS, caudal regression syndrome; M, male; F, female; C, Caucasian; UN, unknown; NA, not available.

The *FZD6* p.Arg405Gln (c.1214G>A) was found in two unrelated patients both affected with myelomeningocele (Figs. 1 and 2; Table 2). This variant was detected in the father of the Italian patient who represents a NTD familial case with an affected third-degree relative. The mutation affects a conserved residue predicted to localize to the third intracellular domain of the protein and changed a positively charged residue into a hydrophilic uncharged residue (Fig. 1C). Although PANTHER and PolyPhen predicted that this variant is benign, DomPred software showed an effect on the secondary protein structure. Moreover, the identification of this variant in two unrelated patients affected with the same kind of open NTDs and its absence in all controls strongly suggests that it is most likely pathogenic.

The *FZD6* p.Arg511His (c.1532G>A) was found in an Italian patient with Caudal Regression syndrome, including vertebral and costal anomalies, meningocele, hydromyelia, tethered cord, left kidney agenesis (Figs. 1 and 2; Table 2); it is predicted to localize to the cytoplasmatic terminal domain of the protein (Fig. 1B). The mutation was transmitted from his apparently healthy mother. Although the variant represents an exchange of two basic amino acids that is not considered to be a substantial chemical change, these residues do lie within an absolutely conserved region suggesting intolerance for any amino acid substitution across evolution (Fig. 1C).

The *FZD6* p.Arg511Cys (c.1531C>T) was identified in an Italian patient with myelomeningocele (MMC) (Figs. 1 and 2; Table 2). This substitution introduces a cysteine in an absolutely conserved region and may result in the formation of intermolecular disulfide bridges leading to abnormal conformational changes of the FZD6 protein (Fig. 1C). The identification of two different missense mutations, p.Arg511His and p.Arg511Cys, affecting the same conserved amino acid in two unrelated NTD patients demonstrate that this residue could represent a mutational hot spot. Both transitions affect a CG dinucleotide, therefore the sequence predisposes to mutations. Moreover, missense mutations at this site could confer a specific pathogenic effect as supported by bioinformatic analysis.

The *FZD6* c.^*^20C>T localizes at the 3′ UTR of the gene, outside of protein coding sequence. It was identified in an Italian patient with MMC (Fig. 1A; Table 2). The C→T substitution altered the predicted target site for MIR628 (hsa-miR-628-3p; miRNA binding score = −0.6; cutoff score is < −0.1), suggesting that this "apparent silent" variant may have an effect creating aberrant cis-regulatory element for miRNA (Table 1).

In silico analysis predicted that *FZD6* p.His140Tyr (c.418C>T; rs80216383:C>T) and p.Ala388Asp (c.1163C>A) mutations, even if they were detected in one and two of 639 controls, respectively, could affect protein function, demonstrating that these variants could represent predisposing risk factors (Table 1). Intriguingly, the *FZD6* p.His140Tyr variant occurred in cis with two benign missense mutations, *FZD6* p.Gly604Arg (c.1810G>A; rs79408516:G>A) and *FZD6* p.Ser620Thr (c.1858T>A; rs116195528:T>A) (Table 1).

## Discussion

This is the first study that systematically screened all *FZD3* and *FZD6* coding regions and splice sites for small alterations in patients with NTDs. Resequencing analysis in 473 NTD patients and 639 controls identified a statistically significant overrepresentation of rare presumed pathological variants among the patients group. Prediction and understanding of the downstream effects of the nonsynonymous and noncoding variants was done using computational methods. Five mutations of *FZD6*, one de novo nonsense mutation (*FZD6* p.Cys615X), three missense variants (*FZD6* p.Arg405Gln, p.Arg511Cys, p.Arg511His) affecting highly conserved residues, and a 3′UTR substitution (*FZD6* c.^*^20C>T) were absent in all controls analyzed and have detrimental effects on the basis of bioinformatic tools. Thus, our findings support a significant involvement of *FZD6* in the pathogenesis of a minority of NTDs patients and underscore the value of candidate gene resequencing to understand the genetic contribution in complex diseases such as NTDs. NTDs are the second human disease to be linked to mutations of *FZD* genes after familial exudative vitreoretinopathy (FEVR; MIM# 133780) that arise from loss of function mutations of *FZD4,* a FZD gene that has not far been implicated in neural tube closure [Robitaille et al., 2002].

*FZD6* mutations account only for 1% of the NTDs cases. This mutation frequency is comparable to that detected in our previous studies in other human PCP genes [Kibar et al., 2009, 2010]. The majority of the mutations were inherited from a healthy parent demonstrating incomplete penetrance. This finding suggests that mutations at *FZD6* gene represent low penetrance variants that must interact with other genes and/or environmental factors to modulate the incidence and the severity of NTDs. All the unique mutations were heterozygous and private except for *FZD6* p.Arg405Gln found in two unrelated patients: one Italian familial case and one sporadic Canadian patient. The majority of the mutations were located in the intracellular domains of the FZD proteins. Mutagenesis studies have demonstrated that several residues in the first and third intracellular loops as well as in C-terminal domain of FZD are crucial for signaling [Cong et al., 2004].

Importantly, the *FZD6* p.Cys615X arises from an A insertion in the coding region, which introduces a premature stop codon resulting in a truncated protein lacking the last 51 residues in its C-terminal tail. Genotype–phenotype correlation shows that this de novo and severe *FZD6* mutation is associated with a complex dysraphic state in a patient presenting with thoracic meningocele, spondylo-costal malformations, hydromyelia, and lipoma. This is the first frameshift truncating mutation reported in PCP genes, since all previous reported mutations were missense [Kibar et al., 2007, 2009, 2011]. More remarkably, the *FZD6* p.Cys615X is a new germinal event, strongly implicating that this mutation might contribute to the NTD occurrence in this patient.

Mutations in the 3′ UTR of mRNAs can lead to the removal or to the de novo generation of a target recognition site for a specific miRNA [Ambros, 2004]. miRNAs are implicated in a wide range of basic biological processes, including development, differentiation, apoptosis, and proliferation [Bartel, 2004]. There are now some examples of sequence variations in the 3′ UTR of mRNAs altering miRNA recognition sites, which have been suggested to have a pathogenic role in human genetic diseases [Lu et al., 2008]. Our present data of a novel predicted altered miRNA binding site of *FZD6* gene in a patient with myelomeningocele adds to the already complex picture of NTDs pathogenesis.

Unique nonsynonymous *FZD6* mutations were detected both in open and closed spinal NTDs. This suggested that *FZD6* gene could contribute in the pathogenesis of both open and closed forms of NTDs, as reported for other PCP genes, *VANGL1* and *VANGL2* [Kibar et al., 2007, 2009, 2010], further supporting a common mechanism involving defective PCP genes in the onset of both NTDs forms.

By contrast, we could not demonstrate a significant contribution of *FZD3* gene in the pathogenesis of NTDs. In fact, only one mutation was identified having an evident pathogenetic effect on protein function. Further studies would be required to determine whether *FZD3* may play an independent role.

Mouse models represent powerful tools for gene discovery in human disease. *Fzd3*^−/−^/*Fzd6*^−/−^ mice mutants provided a successful entry point for identifying human orthologs involved in NTDs. However, comparison of mouse and human data provide some limited insights, in that differently from double mutant mice that develop a severe form of NTDs, mutations of both *FZD* are unlikely to be found in the same affected individuals, probably due to their lethality. In addition, as reported in mice [Wang et al., 2006a], we confirmed a redundant function of *FZD3* and *FZD6* genes in neural tube closure, since that mutations at one single gene are not sufficient to develop the defect, being the majority of them inherited from an healthy parent, probably because of a compensatory effect of the wild-type gene. Given that single-locus allelism is insufficient to explain the variable penetrance and expressivity of such malformations, genetic variation across multiple sites of the PCP proteome may occur to influence clinical outcome.

In conclusion, our findings are consistent with the emerging model that the cumulative contributions of multiple rare alleles with large genetic effects are found among individuals with complex trait. Identification of the role of *FZD6* gene in NTD represents a further step forward to our understanding of the intricate genetic puzzle underlying these complex malformations. Functional studies of the *FZD6* variants will be necessary to confirm their implication in NTDs risk. Finally, our promising results induce us to pursue the investigation of other core PCP genes in large human NTDs cohorts.
